# Australian Notes

**Published:** 1910-01-15

**Authors:** 


### AUSTRALIAN NOTES.

In this year's State Budget, £104,000 is voted for charities, and it is also part of the Government programme to erect a new and modern building at a cost of £200,000 for the treatment of the insane.

It is reported from the Pacific that leprosy is steadily increasing in the northern Cook Islands, there being at the present time thirty known cases, while on the island of Penrhyn the percentage is 1 in 15 of the whole population.

A beginning has been made in Victoria with the medical inspection of school children. The announcement was made early in August by the Minister for Education (Mr. A. A. Billson) that three medical officers would be appointed, and before the end of the month thirty-three applications for the positions had been received, of which eight were from women. The daily newspapers of September 25 notified that the State Public Service Commissioner had appointed: 1, Dr. Harvey Sutton, first (Victorian) Rhodes Scholar; 2, Dr. Mary Booth; and 3, Dr. Jean Greig as officers to inaugurate the system here. The salaries carried are: 1, £550; 2, £500; and 3, £450 per annum.

The death of Dr. Philip Edward Muskett, a voluminous writer upon diet and the feeding and management of infants and children, took place in Sydney at the age of fifty-two years, from an affection of the heart. Dr. Muskett was born at Melbourne, and did the five years' medical course at the University, then went to Glasgow and Edinburgh Universities to see what the old world did. On his return to Australia he was appointed Resident Medical Officer to the Melbourne Hospital. Later —1882—he held the same position in the Sydney Hospital. For over twenty years he has been in private practice, but has held the offices of Surgeon Superintendent to the New South Wales Government and Medical Superintendent of the Sydney Quarantine Station. Dr. Muskett's views upon the value of diet and the mode of bringing children up are embodied in nearly a dozen books, several of which include excellent recipes for salads and other light French or Italian dishes, which he maintained were more suitable to the Australian climate than the usual stodgy British diet commonly followed. Dr. Muskett's estate, about £15,000, is left to his sister for life, but after her death, with a number of other bequests, sums are to go to the Children's Hospitals of Australia and New Zealand, and the Philip Muskett Biennial Bequest is to be created, to be competed for in the form of an essay, every other year, with the object of promoting the principles which the testator so strongly advocated in all his published works. A favourite quotation of Dr. Muskett's was this of Lord Beaconsfield's: "The fate of a nation will ultimately depend upon the strength and health of the population."

Speaking to the National Council of Women of Queensland (affiliated with the International Council of Women, of which the Countess of Aberdeen is President) in Brisbane in August, her Excellency the Countess of Dudley gave a speech of which the main part was as follows:—

"Two suggestions have come to me from what I have seen in the country districts of Queensland and elsewhere, and I mention them to-night because if they commend themselves to you it seems to me they are peculiarly part of the work which women do best and for which they are most fitted. The first of these is district nursing—not only town district nursing, but country district nursing as well. I know this whole question of country district nursing in Australia is surrounded by many and great difficulties, and even were a practical scheme formulated it would not be easy to carry it out. But few reforms which need a widespread organisation are easy of accomplishment, and with courage, common sense, and perseverance difficulties which appear insuperable are eventually overcome. The question I would desire to see considered by the women of Australia is that if the hardship entailed upon women and children in times of accident or illness, who, living in remote bush districts, are far removed from medical or skilled assistance; if it were an established fact that to such as these the ministrations of a trained nurse resident in the district would be a boon and an assistance, then we need only concern ourselves with the recognition of this fact, and set to work to meet the need regardless of all difficulties in the way."

No need to ask if trained nurses are not needed, and would not be greatly appreciated in the Bush! We all recognise the need. But those best acquainted with the vast distances of our country and its unpeopled condition felt their breath taken away at the audacity of the proposition. Yet it is a fact that bush isolation is to some extent mitigated by a fairly good telegraph system; that the telephone service is extending; and that, above all modern inventions, the motor-car seems as if it had been specially created for use in Australia. Already many squatters use it for going over their runs as well as for long journeys. No sooner was Lady Dudley's suggestion made public than offers of help were forthcoming. An anonymous donor has offered twenty acres of land on a main road in Victoria as site for a nurses' home or a hospital, and Madame Melba, who is in her native country at present, at once offered to give a concert—for the nucleus of the fund, without which nothing can be done—before she leaves Australia, has issued an appeal to all women, and offered prizes to those who collect most money.

